# Perturbation of the metabolic network in *Salmonella enterica* reveals cross-talk between coenzyme A and thiamine pathways

**DOI:** 10.1371/journal.pone.0197703

**Published:** 2018-05-23

**Authors:** Dustin C. Ernst, Andrew J. Borchert, Diana M. Downs

**Affiliations:** Department of Microbiology, University of Georgia, Athens, Georgia, United States of America; Universite Paris-Sud, FRANCE

## Abstract

Microorganisms respond to a variety of metabolic perturbations by repurposing or recruiting pathways to reroute metabolic flux and overcome the perturbation. Elimination of the 2-dehydropantoate 2-reductase, PanE, both reduces total coenzyme A (CoA) levels and causes a conditional HMP-P auxotrophy in *Salmonella enterica*. CoA or acetyl-CoA has no demonstrable effect on the HMP-P synthase, ThiC, *in vitro*. Suppressors aimed at probing the connection between the biosynthesis of thiamine and CoA contained mutations in the gene encoding the *ilvC* transcriptional regulator, *ilvY*. These mutations may help inform the structure and mechanism of action for the effector-binding domain, as they represent the first sequenced substitutions in the effector-binding domain of IlvY that cause constitutive expression of *ilvC*. Since IlvC moonlights as a 2-dehydropantoate 2-reductase, the resultant increase in *ilvC* transcription increased synthesis of CoA. This study failed to identify mutations overcoming the need for CoA for thiamine synthesis in *S*. *enterica panE* mutants, suggesting that a more integrated approach may be necessary to uncover the mechanism connecting CoA and ThiC activity *in vivo*.

## Introduction

There is a growing appreciation for the ability of metabolic perturbations to impact points in the metabolic network that seem to be distantly, or not at all, connected [1–3]. The subtle connections between pathways that mediate these effects can often be enhanced through mutant analysis, which leads to a better understanding of the underlying structure and cross-talk of metabolic networks. For example, constraining an essential metabolic pathway requires that a cell overcome the restriction or risk cell death. The mechanisms available to overcome perturbations in the metabolic network can reveal inherent robustness in the system, and further uncover connections between biochemical processes that could not be predicted from prior knowledge. Mutant analysis probing the cellular responses to metabolic perturbations has uncovered a variety of emergent mechanisms of thiamine biosynthesis in bacteria [4, 5].

**Fig 1:**
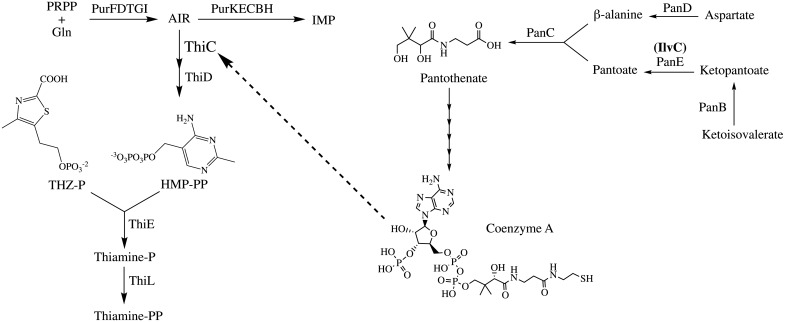
Thiamine and coenzyme A biosynthesis. (*Left*) The thiamine biosynthetic pathway uses the branch-point metabolite aminoimidazole ribotide (AIR) from *de novo* purine biosynthesis. ThiC catalyzes formation of HMP-P from AIR, which is subsequently phosphorylated prior to being condensed with THZ-P to form thiamine-phosphate. Thiamine-phosphate is further phosphorylated to thiamine-pyrophosphate (TPP). (*Right*) Coenzyme A is synthesized from pantothenate in five steps. The production of the pantoate is predominantly catalyzed from ketopantoate, an intermediate in valine biosynthesis, by PanE. The branched-chain amino acid biosynthetic enzyme acetohydroxyacid isomoreductase (IlvC) has weak ketopantoate reductase activity.

The biosynthetic pathway for coenzyme A (CoA) shares several features with that of thiamine biosynthesis. Specifically, each pathway produces an essential nutrient, and the flux of carbon and energy required for both products is significantly lower than that of the pathways they derive precursors from (purine and branched chain amino acids, respectively) (Fig 1) [4, 6]. A lesion eliminating the primary 2-dehydropantoate 2-reductase in *Salmonella enterica*, PanE (EC: 1.1.1.169), results in severe reduction in total CoA levels, but does not generate a supplementation requirement for growth [7]. The residual production of CoA in the *panE* mutants is due to the activity of a redundant 2-dehydropantoate 2-reductase activity inherent in ketol-acid reductoisomerase (IlvC; EC: 1.1.1.86), an enzyme required for branched-chain amino acid biosynthesis [8]. Thus, a *panE* mutant maintains ~ 10% of wild type CoA levels, which is sufficient for prototrophic growth [7].

Previous work established a link between intracellular CoA levels and thiamine biosynthesis (Fig 1). Thiamine pyrophosphate is an essential cofactor, and is made of two independently synthesized moieties, 4-methyl-5-(2-hydroxyethyl)-thiazole phosphate (THZ-P) and 4-amino-5-(hydroxymethyl)-2-methylpyrimidine phosphate (HMP-P). Mutations in *panE* not only reduce CoA levels in the cell, but also cause a conditional HMP-P auxotrophy in *S*. *enterica* [9, 10]. Disruption of *panE* leads to a requirement for exogenous HMP when elimination or inhibition of amidophosphoribosyltransferase (PurF; EC: 2.4.2.14) compromises flux through the shared purine/HMP-P biosynthetic pathway [7, 9]. Purines repress expression of *purF* and allosterically inhibit the PurF enzyme, generating a thiamine requirement when *panE* mutants are grown in the presence of adenine [7, 11–13]. Low CoA levels compromise the conversion of AIR to HMP-P, resulting in a requirement for exogenous HMP [14]. The conversion of AIR to HMP is catalyzed by HMP-P synthase (ThiC; EC: 4.1.99.17) [15, 16]. ThiC is a member of the radical *S*-adenosylmethionine (SAM) superfamily of enzymes that use a [4Fe-4S] cluster to initiate radical catalysis [15, 16]. Neither CoA or acetyl-CoA have a demonstrable effect on ThiC activity *in vitro* [17], and the connection between CoA levels and ThiC activity *in vivo* is unresolved.

This study was initiated to probe the connection between the biosynthesis of thiamine and CoA. To gain insights about the role of CoA in the ThiC reaction, suppressor mutations that restored thiamine synthesis to strains lacking *panE* were sought. The suppressors isolated were mutations in the *ilvC* transcriptional regulator, *ilvY* that resulted in an increase in *ilvC* transcription, suggesting the need for CoA had not been bypassed, but the synthesis of this cofactor was simply increased.

## Materials and methods

### Bacterial strains, media and chemicals

All strains used in this study are derivatives of *Salmonella enterica* serovar Typhimurim LT2 and are listed with their genotype in Table 1. Tn*10d*(Tc) refers to the transposition defective mini-Tn*10* (Tn*10*Δ16 Δ17) described by Way et al. [18]. MudJ refers to the Mu*d*1734 transposon described previously [19]. Tn*10*(Cm) refers to the transposition defective Tn*10* specifying chloramphenicol resistance [20].

**Table 1:** Bacterial strains and primers.

Strain	Genotype
LT2 DM3547	Wild type *panC*617::Tn10d(Tc)
DM13650	zxx-8029::Tn10d(Tc) *panE*::Cm
DM13651	zxx-8029::Tn10d(Tc) *thiC*1128 *panE*::Cm
DM13652	zxx-8029::Tn10d(Tc) *thiC*1129 *panE*::Cm
DM13892	zxx-8029::Tn10d(Tc) *thiC*1128 *panE*::Cm *ilvY*3215
DM13896	zxx-8029::Tn10d(Tc) *thiC*1129 *panE*::Cm *ilvY*3214
DM13897 DM13931	zxx-8029::Tn10d(Tc) *thiC*1129 *panE*::Cm *ilvY*3213 zxx-8029::Tn10d(Tc) *thiC*1129 Δ*panE*
DM13956	zxx-8029::Tn10d(Tc) *thiC*1129 Δ*panE* zxx-3911::Tn*10*(Cm)
DM13957 DM13963	zxx-8029::Tn10d(Tc) *thiC*1129 Δ*panE* zxx-3911::Tn*10*(Cm) *ilvY*3213 zxx-8029::Tn10d(Tc) *thiC*1129 Δ*panE ilvY*3213
DM13993	zxx-8029::Tn10d(Tc) *thiC*1129 Δ*panE* / pBAD24
DM13994	zxx-8029::Tn10d(Tc) *thiC*1129 Δ*panE* / pBAD24-*ilvC*
DM13995	zxx-8029::Tn10d(Tc) *thiC*1129 Δ*panE* / pBAD24-*ilvY*
DM13996	zxx-8029::Tn10d(Tc) *thiC*1129 Δ*panE* / pBAD24-*ilvY*3213
Primer	Sequence
STM4195_NcoI_F	5’-GAGACCATGGCCATGCTCGCCG TCATTACC-3’
STM4195_XbaI_R	5’-GAGATCTAGATTAATTTACCTTTGCCGTTT-3’
*ilvC*_NcoI_F	5’-GAGACCATGGCCATGGCTAACTACTTTAAT-3’
*ilvC*_XbaI_R	5’-GAGATCTAGATCAACCCGCTACCGCAATAC-3’
LT2 *gyrB* qRT-PCR 5’	5’-CCGTTGGATCACGAGTTTG-3’
LT2 *gyrB* qRT-PCR 3’	5’-AACGCGTCCTCTTCAATCAG-3’
LT2 *rpoB* qRT-PCR 5’	5’-AGTCGACCTGAGCACCTTCA-3’
LT2 *rpoB* qRT-PCR 3’	5’-CAAACACTGGTGTGGCAATC-3’
LT2 *ilvC* qRT-PCR 5’	5’-CTGTCCGAACAGCTGAAAGAG-3’
LT2 *ilvC* qRT-PCR 3’	5’-GTTAGCCCAGTCAGCCATCA-3’

No-carbon E medium (NCE) supplemented with 1mM MgSO_4_ [21], trace minerals [22] and glucose (11mM) as the sole carbon source was used as minimal medium. Difco nutrient broth (NB) (8g/liter) with NaCl (5g/liter) or Luria-Bertani broth (LB) were used as rich media when indicated. Difco BiTek agar (15g/L) was added for solid medium. When necessary the branched-chain amino acids leucine, isoleucine and valine (and pathway intermediate ketoisovalerate) were added at a final concentration of 0.3 mM. Antibiotics were added to the final concentrations indicated in rich and minimal medium, respectively: tetracycline 20 and 10 μg/ml; ampicillin 30 and 15 μg/ml; and chloramphenicol 20 and 5 μg/ml. When needed, arabinose was added to cultures at 0.1% or 1%, as indicated.

### Genetic methods

All transductional crosses were performed using the high-frequency general transducing mutant of bacteriophage P22 (HT105/1, *int*-201) [23]. Methods for performing transductional crosses, purification of transductants from phage, and the identification of phage-free recombinants have been described previously [9, 24]. All mutant strains were constructed using standard genetic techniques. Gene replacements were made using the λ-Red recombinase system described by Datsenko and Wanner [25].

#### Mutant isolation

Five cultures each of DM13651 (zxx-8029::Tn10d(Tc) *thiC*1128 *panE*::Cm) and DM13652 (zxx-8029::Tn10d(Tc) *thiC*1129 *panE*::Cm) were grown overnight in NB medium. Cells were pelleted and resuspended in an equal volume of saline solution (85mM). Approximately 10^8^ cells from each cell suspension were spread onto solid minimal glucose medium. DES (5 μl) was spotted in the middle of plates containing DM13652. Plated cultures were incubated at 37°C for ~72 hours. Resulting colonies were streaked on non-selective medium (NB), single colonies were patched to rich medium and after ~6 hours of incubation replica-printed to selective (minimal) medium. A representative mutant strain displaying robust growth on minimal glucose medium, DM13897 (zxx-8029::Tn10d(Tc) *thiC*1129 panE::Cm *ilvY*3213) was used to map the causative lesion using standard genetic techniques. Briefly, the chloramphenicol marker was resolved [25] and a pool of ~60,000 cells with random Tn*10d*-Cam insertions throughout the chromosome was generated with the resulting chloramphenicol-sensitive strain (DM13963). A P22 lysate grown on this pool and standard genetic approaches identified Tn*10d*-Cam insertions linked to the causative suppressor mutation. The site of insertion was determined by sequence analyses using degenerate primers and those specific to the Tn*10d*-Cam [26]. DNA sequence was obtained at the University of Wisconsin Biotechnology Center. Transductional crosses confirmed that the Tn*10d*-Cam linked to the causative suppressor mutation in DM13897 was similarly linked to two additional suppressors described here.

### Phenotypic analysis

#### Growth curves

Strains were grown in NB broth overnight. Cultures were pelleted and resuspended in an equal volume of saline. Aliquots (5 μl) of the cell suspension were used to inoculate 200 μl of the desired medium contained in each well of a 96-well microtitre plate. Cultures were grown at 37 °C while shaking at 250 rpm using the Biotek EL808 ultra microplate reader. Cell density was measured as the absorbance at 650 nm. The specific growth rate was determined as [u = ln(*X/X*_0_)/*T*], in which X_0_ is the initial optical density during the linear, *X* is the final optical density during the linear growth phase and *T* is time.

#### Soft agar overlays

DM3547 (*panC*) was grown to full density in NB at 37°C. After incubation, cells were pelleted and resuspended in an equal volume of sterile saline. 100 μl of the saline suspension was added to 3 ml of molten 0.7% agar and spread over a minimal glucose plate containing 50 nM thiamine. The overlay was allowed to solidify 1 h and then a single colony for strains to be tested were stab inoculated through the soft agar overlay and onto the solid media underneath. The plate was allowed to grow 18 h at 37°.

### Molecular methods

The *ilvY* and *ilvC* genes were amplified by PCR with Herculase II Fusion DNA polymerase (Agilent). Primers used to amplify *ilvY* or *ilvC* were *ilvY*_NcoI_F and *ilvY*_XbaI_R or *ilvC*_NcoI_F and *ilvC*_XbaI_R, respectively (Table 1). The resulting PCR products were gel purified, digested with *Nco*I (Promega) and *Xba*I (Promega) and ligated into *Nco*I/*Xba*I-cut pBAD24 [27]. Constructs were transformed into *Escherichia coli* strain DH5α and screened for vectors containing inserts. Plasmid inserts were confirmed by sequencing. Plasmids containing the appropriate insert were purified and transformed into the relevant strains.

## qRT-PCR

RNA from four independent replicates of each *S*. *enterica* strain tested, as described in the text, was extracted. Strains were grown overnight with shaking at 37°C in 2 ml NB. Cultures were then pelleted and resuspended in equal volume of 100 mM saline solution before being diluted 1:100 into 5 ml fresh minimal glucose (11 mM) medium containing 50 nM thiamine and allowed to grow at 37°C with shaking to an OD_650_ = 0.6. RNA was prepared as described previously, using the RNAsnap^™^ method [28, 29]. Briefly, total RNA was extracted at 95°C, using 95% [vol/vol] RNA-grade formamide, 18 mM EDTA, 0.025% [wt/vol] SDS, 1% 2-mercaptoethanol in UltraPure^™^ (ThermoFisher) distilled water. RNA was then treated with RNase-free Turbo DNase (Ambion). Samples were precipitated by sodium acetate-ethanol precipitation and resulting RNA was stored at −80°C.

The University of Georgia Genomic Facility (GGF) assessed the samples for quality and quantification using the RNA 6000 nano kit for the Agilent 2100 bioanalyzer. qRT-PCR preparation and analysis methods are described elsewhere [29]. Samples with an RNA integrity number (RIN) over 5.0 were used [30]. iScript cDNA synthesis kit (Bio-Rad Laboratories) generated first strand cDNA from 800ng RNA. 20 μl reactions contained 10 μl FastStart Universal SYBR green Master (ROX) mix (Roche Applied Science), 8 ng cDNA, and two gene-specific primers (0.5 μM) (Table 1), and were run on an Applied Biosystems 7500 Fast real-time (RT) PCR system. Real-time cycling conditions were: 95°C for 20 s, and 40 cycles of 95°C for 3 s and 60°C for 30 s. *gyrB* was used as an internal control [31], and fold-change was calculated using the comparative threshold cycle (ΔΔCT) method [32]. Gene expression (mutant/wild-type) fold-change = 2^ΔΔCT^, where ΔΔCT = ΔCT_mutant_ − ΔCT_wild-type_ and ΔCT = CT_target gene_ (*ilvC*) − CT_normalization gene_ (*gyrB*). To ensure *gyrB* expression was constant for all strains, 2^ΔΔCT^ values were calculated against the alternative internal control *rpoB* [33]. In this case, no significant fold-change was detected.

### Coenzyme A quantification

Overnight cultures for the strains analyzed were prepared in NB broth. Cultures were pelleted and resuspended in equal-volume 100 mM saline solution. Culture flasks containing 200 ml of minimal medium with glucose (11 mM) as the sole carbon source, supplemented with 50 nM thiamine, were inoculated to 2% final inoculum. Cells were grown at 37°C while shaking to a final OD_650_ of 0.3, harvested by centrifugation at 8,000 X G for 12 minutes, and stored at -80°C, until ready for analysis. CoA levels were determined by a modified method of the one described by Allred and Guy [34, 35]. Briefly, cells, resuspended in phosphate buffered saline, were lysed through the addition of 0.25 N formic acid and allowed to incubate 30 min on ice, vortexing briefly. The lysate was centrifuged (14,000 X G) for 10 min to remove cell debris and then neutralized by the addition of NH_4_OH (pH = 7.0). Reductive cleavage of CoA thioesters was achieved through addition of dithiothreitol (0.7% [vol/vol]) and quantification of CoA was carried out by coupled enzymatic assay. 100 μL reactions contained 40 μL lysate, 300 μmol Tris (pH 7.2), 60 μmol KCl, 18 μmol malate, 7.2 μmol acetyl-phosphate, 1.2 μmol NAD^+^, 0.4 U citrate synthase, 2 U malate dehydrogenase, and 0.9 U phosphotransacetylase. NADH formation was determined by monitoring absorbance at 340 nm using a Spectramax M2. CoA quantification was made following comparison to a standard curve of known CoA additions. Statistical significance (P < 0.01) was determined by conducting one-way analysis of variance (ANOVA) and Tukey’s post-test using GraphPad Prism for Mac OS X 7.0c.

## Results and discussion

### ThiC variants cause an auxotrophy in a Δ*panE* background

A previous report described ThiC variants (ThiC^E281K^ and ThiC^V267M^) that allowed growth on minimal medium, but were unable to support thiamine-independent growth when adenine was added to the growth medium [36]. The data were consistent with the effect of adenine being to decrease carbon flux through purine biosynthesis, resulting in lower substrate (AIR) concentration for ThiC [37]. The phenotype of strains carrying the respective *thiC* alleles appeared similar to that of strains lacking *panE* in their inability to synthesize thiamine when adenine was present in the medium. Strains containing either of the above ThiC variants and a lesion in *panE* were auxotrophic, even on minimal medium lacking adenine. Significantly, either pantothenate or thiamine supported growth of these strains (Table 2). This finding re-emphasized the connection between CoA and ThiC activity. A simple interpretation of these data was that each mutation (i.e., lack of *panE* or compromised ThiC variant) weakly constrained the HMP pathway and the constraints were additive such that the combination of ThiC^E218K^ or ThiC^V267M^ with a lesion in *panE* prevented growth on minimal glucose medium.

**Table 2:** Growth of *panE* strains containing ThiC variants^a^.

Strain	ThiC variant	Specific Growth Rate			Final Cell Yield		
		-	+ Pant	+ Thi	-	+ Pant	+ Thi
DM13650	ThiC^WT^	0.41	0.67	0.50	0.75	0.84	0.71
DM13651	ThiC^E281K^	0.03	0.67	0.50	0.15	0.89	0.75
DM13652	ThiC^V267M^	0.01	0.70	0.54	0.14	0.88	0.77

^*a*^Strains were grown in NCE medium supplemented with glucose (11 mM) and the indicated additions. Pant, pantothenate; Thi, thiamine. Growth rate is reported as μ = ln(*X*/*X*_0_)/*T*, and the final cell yield is *A*_650_ after 12 h of growth. Data shown are the average of three independent cultures. All standard deviations were less than 0.03.

### Mutations in *ilvY* suppress thiamine requirement

**Fig 2:**
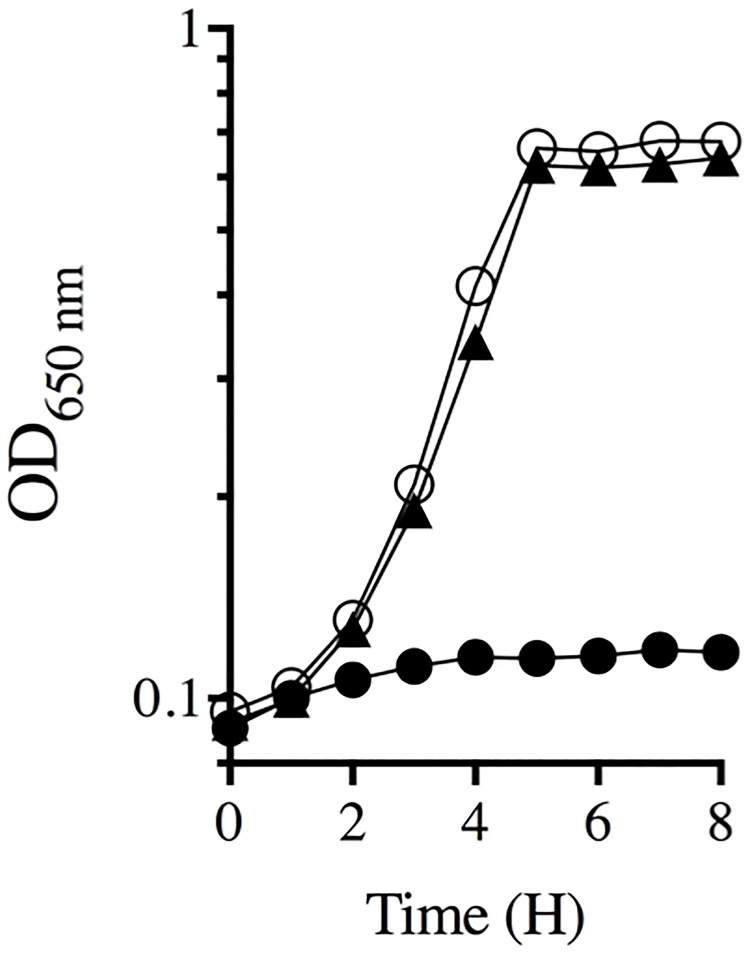
Mutant derivatives of a *thiC1129 panE*::*Cm* strain grow on minimal glucose medium. The parental *thiC panE* strain fails to grow on minimal glucose medium (filled circles), but a suppressor derivative (DM13897) grows well (filled triangles). Growth of the parental strain was restored by the addition of thiamine (100 nm) (open circles) or pantothenate (100 μM). Growth data show representative experiment repeated with three independent cultures.

**Fig 3:**
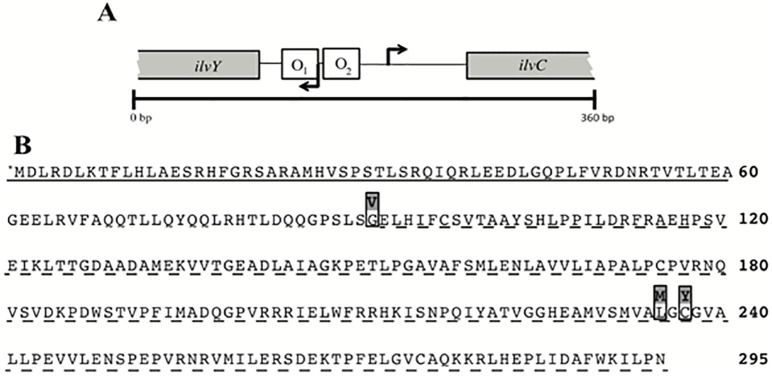
Regulatory region of *ilvY* and *ilvC* and IlvY protein sequence. **(A)** Genetic context of the regulatory region of *ilvY* and *ilvC* in *S*. *enterica* genome is schematically represented. Map is zoomed to include only the first 100 bp of *ilvY* and *ilvC* genes. Arrows denote transcription start site for the respective gene. Binding of *ilvY* product at O_1_ and O_2_ operator sites, in the presence of IlvC substrate, is required for RNA polymerase binding to the *ilvC* promoter [38]. **(B)** Protein alignment of wild-type IlvY with IlvY variants from suppressor mutants. (*M) denotes the start (fMet) of the protein. The solid underlined section indicates the N-terminal helix-turn-helix and the dashed underline section identifies the C-terminal substrate-binding domain. Variant residues in IlvY^G92V^ (*ilvY*3215), IlvY^L235M^ (*ilvY*3213), and IlvY^C237Y^ (*ilvY*3214) are shown above the IlvY protein sequence.

Suppressor analysis was used to probe the role of CoA in ThiC activity and HMP synthesis. Mutations were sought that bypassed the need for CoA and thus restored growth to the *panE thiC* mutant strains on minimal medium. Multiple mutant suppressors were isolated and triaged according to growth phenotypes. Growth of representative suppressor strain DM13897 (*thiC1129 panE*::Cm *ilvY3213*) compared to parental strain DM13652 (*thiC1129 panE*::Cm) in minimal glucose medium is shown in Fig 2. While the suppressor strain grew on minimal glucose medium, the parental strain failed to grow unless either thiamine or pantothenate was provided. The causative mutation in DM13897 was identified as a base substitution in *ilvY*, that changed basepair 703 from a C-to-A and resulted in an L235M variant protein (Fig 3B). Another mutation allowing growth of DM13652 mapped to the same region of the chromosome and was a base substitution in *ilvY*. This strain (DM13896) had a G-to-A transition at basepair 710, corresponding to amino acid change C237V. Finally, a suppressor mutation isolated in strain DM13651, which carried a different *thiC* allele was a G-to-T mutation at basepair 272, generating IlvY variant G92V. The growth rates of these three suppressor mutants were not significantly different from each other (data not shown).

IlvY is a positive regulator of *ilvC; ilvY* is expressed divergently from *ilvC* and the *ilvYC* locus represents a prototypic LysR-type regulated system (Fig 3A) [39]. IlvY binds to the *ilvC* operator but activates transcription only when IlvC substrates (either α-acetolactate or α-acetohydroxybutyrate) are bound as co-inducers [38]. The substitutions in the IlvY variants here are within the effector-binding domain, and to our knowledge represent the first identified substitutions in this domain that are constitutive for expression of *ilvC*. Mutations in *E*. *coli ilvY* that lead to constitutive expression of *ilvC* have been described [40]; however, of these, only the *ilvY2143* allele was further characterized as IlvYG86A [41].

### IlvY variants increase expression of *ilvC*

**Fig 4:**
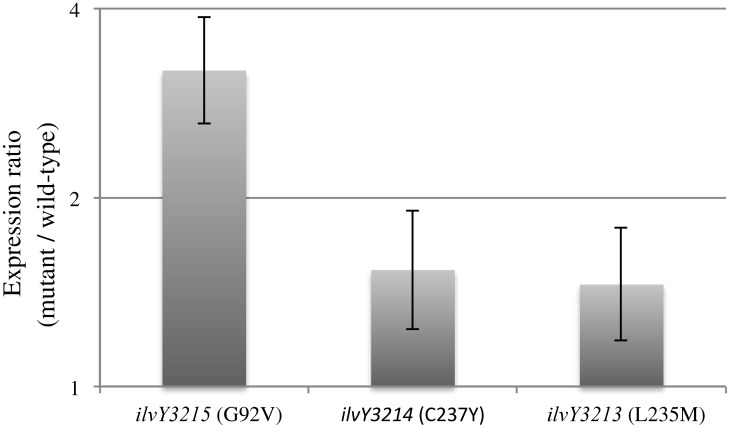
Mutant *ilvY* alleles increased expression of *ilvC*. Differential expression of *ilvC* caused by the indicated *ilvY* alleles is shown. In each case comparison was to the parental strain as described in the text. Strains were grown in minimal glucose medium containing 50 nM thiamine, error bars represent the 95% confidence interval.

Expression of *ilvC* was determined in the *ilvY* mutant strains and the relevant parental strains by Quantitative Real-Time Reverse Transcriptase PCR (qRT-PCR). Strains carrying the *ilvY* alleles *3214* and *3213* (DM13896 and DM13897, respectively) were compared to the parental strain they were derived from, DM13652, and the strain with *ilvY3215* (DM13892) to its parent, DM13651. Transcription of *ilvC* was significantly (p-value < 0.05) increased for each of the *thiC panE* strains harboring mutant *ilvY* alleles (Fig 4). Two points were taken from these data. First, each *ilvY* allele that supported growth of a *thiC panE* mutant increased the expression of *ilvC*, as expected by the position of the substitution in the proteins. Secondly, the two variants that had adjacent substitutions resulted in similar modest increases in *ilvC* transcription, while the more distantly located substitution (G92V) produced a larger, though still modest, increase. Further, the 3-fold increase in *ilvC* expression seen for IlvYG92V was consistent with the ~3-fold increase in *ilvC* expression caused by the nearby and previously characterized G86A substitution in the *E*. *coli* protein [40, 41].

### Expression of *ilvC* or *ilvY* mutants *in trans* restores thiamine synthesis

**Fig 5:**
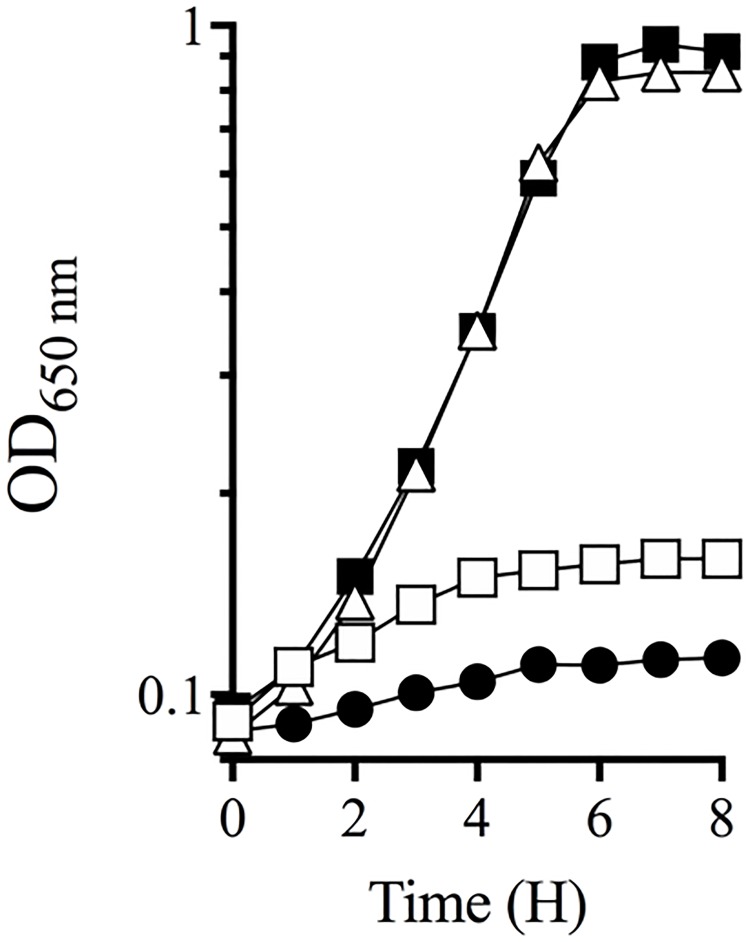
Expression of *ilvY*3213 or *ilvC in trans* restores growth to a *thiC panE* strain. Strains were grown in glucose minimal medium containing 1.0% arabinose. Growth is shown for a *thiC*1129 *ΔpanE* strain containing empty vector, pBAD24 (DM13993; open squares), pBAD24-*ilvY* (DM13995; solid circles), pBAD24-*ilvC* (DM13994; open triangles), and pBAD24-*ilvY*3213 (DM13996; solid squares) as a function of time. Data are representative of three independent cultures.

The qRT-PCR data suggested that the suppressing effect of the *ilvY* alleles was simply due to increased IlvC activity. Consistently, expression of *ilvC in trans* restored growth to a thiamine-limited *panE thiC*1129 strain (DM13994) on minimal glucose medium (Fig 5). Similarly, when a representative *ilvY* allele (*ilvY*3213) was expressed *in trans*, growth was restored to the *panE thiC*1129 mutant on minimal glucose medium (Fig 5). Importantly, expression of wild-type *ilvY in trans* failed to restore growth to the same strain. These data showed that *ilvY*3213 was dominant. Further, the phenotypic similarity achieved by expressing *ilvC* or *ilvY*3213 *in trans* is consistent with increased *ilvC* expression being necessary and sufficient to restore thiamine synthesis to the *panE thiC* mutant strains used herein.

### Suppressed strains have increased CoA pool size

**Fig 6:**
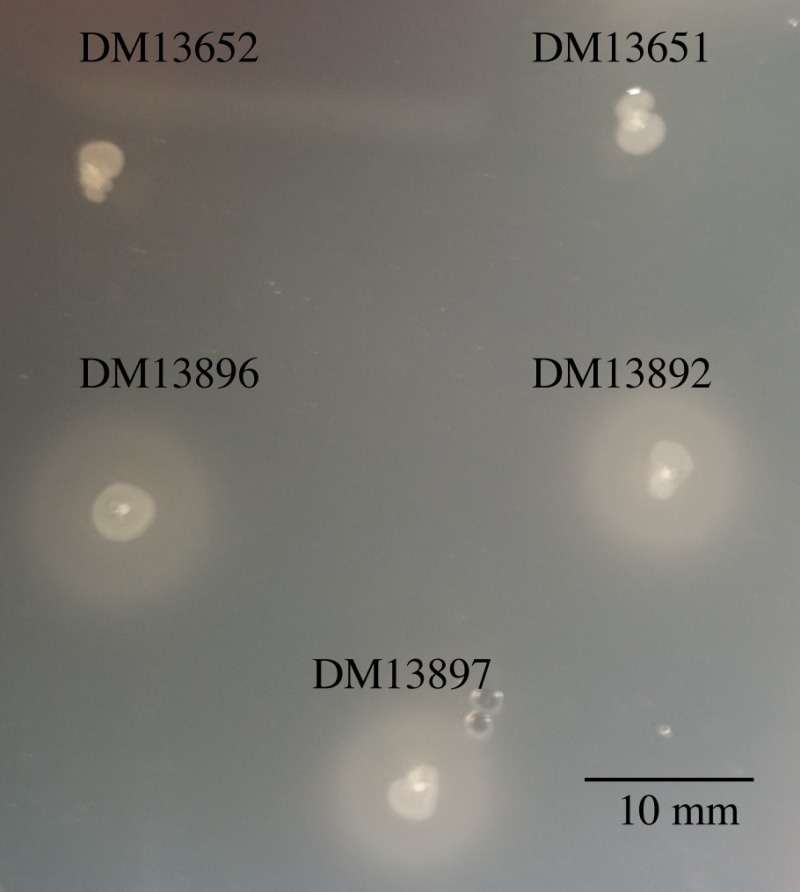
*ilvY* variants feed a pantothenate auxotroph. DM3547 (*panC*) was overlaid (in soft agar) onto minimal glucose medium containing 50 nM thiamine. Single colonies of DM13651 (*thiC*1128 *panE*), DM13652 (*thiC*1129 *panE*), DM13892 (*thiC*1128 *panE ilvY*3215), DM13896 (*thiC*1129 *panE ilvY*3214), and DM13897 (*thiC*1129 *panE ilvY*3213) were stab inoculated into the medium and allowed to grow at 37°C 18 h.

The increased transcription of *ilvC* caused by the isolated alleles of *ilvY* suggested suppression could be due to increased pantothenate, and thus CoA, levels allowed by the 2-dehydropantoate 2-reductase activity of IlvC [8]. The relevant strains were assessed for their production of pantothenate and pantoate using feeding assays. Colonies of the *ilvY* mutant strains were stabbed into a soft agar overlay, containing an embedded *panC* mutant (DM3547), grown on solid glucose minimal medium containing thiamine. Thiamine was added to allow growth of the parental *thiC*1128 *panE* and *thiC*1129 *panE* strains; previous work suggests that thiamine abundance does not directly alter CoA levels in the cell [7]. All three *ilvY* strains excreted more pantothenate than the parent from which they were derived (Fig 6). Similar results were found when pantoate was queried (data not shown). These data further suggested the CoA levels would be elevated in the suppressor mutants.

**Fig 7:**
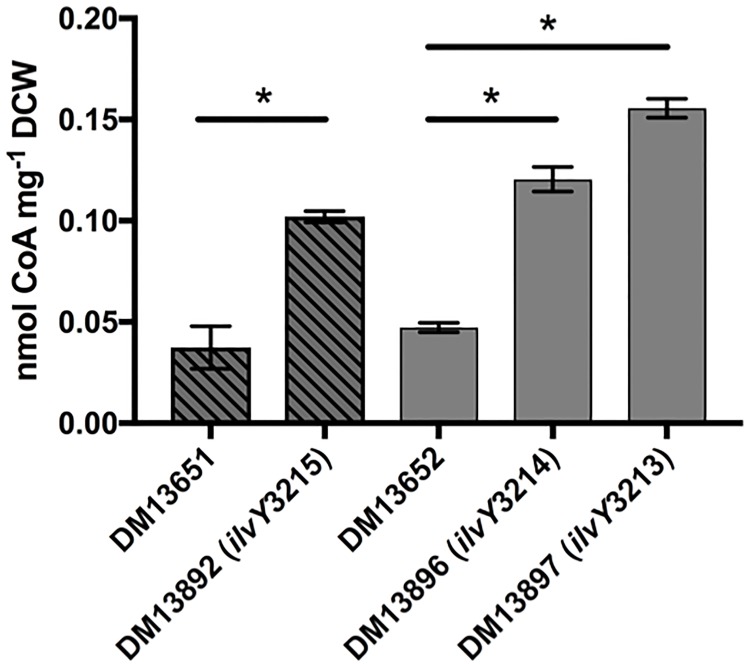
*ilvY* variants increase endogenous CoA levels. Total CoA levels were measured in cells grown in minimal glucose medium with 50 nM thiamine. The data from three independent cultures are represented as the average and standard deviation with an * denoting statistical significance (P < 0.01).

**Fig 8:**
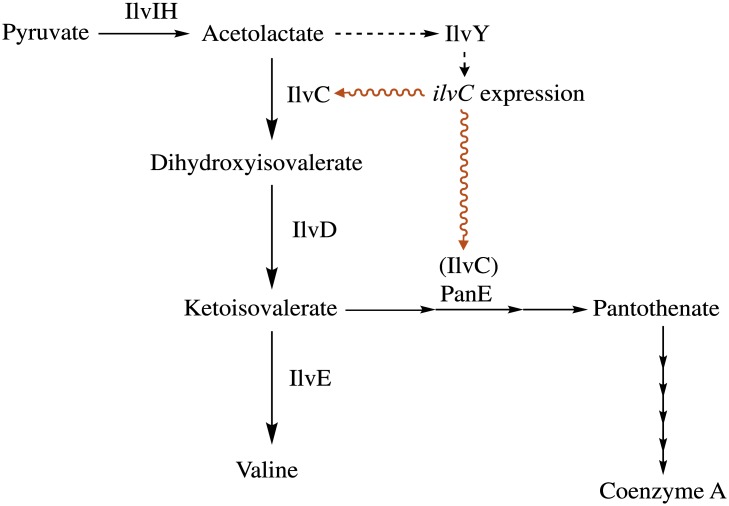
Model of suppression of thiamine requirement mediated by *ilvY* alleles. The IlvY variants described here activate expression of *ilvC*, enhancing ketopantoate reductase activity in the cell, leading to increased CoA levels that improve ThiC-variant activity to restore thiamine production. The mechanism by which CoA increases ThiC activity remains unclear.

Total intracellular CoA levels were assessed in the parental and suppressor strains (Fig 7). In all cases, the strains with the *ilvY* alleles had 2- to 3-fold more Coenzyme A, than the appropriate parental strain. Given these data, we propose the model depicted in Fig 8, where mutations in the *ilvY* effector-binding domain produce constitutively active variants capable of inducing expression of *ilvC*, even in the absence of co-inducer molecules. The constitutive expression of *ilvC* increases IlvC-dependent 2-dehydropantoate 2-reductase activity, leading to improved CoA biosynthesis in the *panE* strain background. Improved CoA biosynthesis in turn results in improved conversion of AIR to HMP-P, restoring thiamine synthesis and growth in the absence of supplementation [14].

### Conclusions

Multiple studies have shown that the conversion of AIR to HMP by ThiC in *S*. *enterica* is sensitive to the level of CoA in the cell [7, 14]. The reason that CoA levels can impact the activity of ThiC *in vivo* has not been defined, and CoA is not required for the enzyme to turn over *in vitro* [17]. The genetic selection described here, as well as others, have failed to identify a class of mutations that overcome the need for CoA in this essential conversion. In the absence of such mutants, efforts to dissect the connection between CoA and thiamine biosynthesis *in vivo* must focus on the metabolic network structure and how CoA functions in defining this structure. The integrated use of diverse approaches is most likely to uncover the mechanism that connects CoA and the activity of ThiC *in vivo*.

LysR proteins, including IlvY, represent the most common type of transcriptional regulator in prokaryotes [39]. The binding of IlvY to the *ilvYC* promoter region negatively auto-regulates transcription of *ilvY*, and upon binding of a coinducer molecule (α-acetolactate or α-acetohydroxybutyrate), activates transcription of the *ilvC* gene. Importantly, the binding of coinducer does not alter IlvY DNA-binding affinity, but rather, induces a conformational change in the preformed IlvY-DNA complex that promotes transcription of *ilvC*. The substitutions in IlvY described here occur in or near the effector binding domain, indicating that the variant IlvY proteins likely bind the *ilvYC* promoter and induce an immediate conformational change in the IlvY-DNA complex irrespective of coinducer molecule binding. These findings are consistent with previous reports that describe substitutions in LysR protein effector-binding domains that lead to constitutive activation of the respective target genes [42, 43]. A protein structure for IlvY has yet to be determined, limiting our ability to assess the mechanistic impact of the amino acid substitutions on IlvY output activity. Future structural characterization of the IlvY variants described here, coupled with the existing robust *ilvYC* operon molecular data, may improve our understanding of LysR protein function. Improved mechanistic understanding of LysR-type transcriptional regulators is likely to impact models of virulence gene expression [44] and may lead to improved metabolic engineering strategies used for microbial chemical production [45].
